# Influence of Thickness of Opaque Porcelain and Alloy Color on the Final Color of Porcelain-Fused-to-Metal Restorations

**DOI:** 10.3390/ma16010457

**Published:** 2023-01-03

**Authors:** Alessandro Vichi, Gabriele Corciolani, Michele Carrabba, Alvaro Munoz, Chris Louca

**Affiliations:** 1 Dental Academy, University of Portsmouth, Portsmouth PO1 2QG, UK; 2 Private Practice, 58100 Grosseto, Italy; 3 Private Practice, 53100 Siena, Italy; 4 Faculty of Dentistry, University of Chile, Santiago 8380544, Chile

**Keywords:** color, opaque, ceramics, PFM, alloy

## Abstract

Despite the advent of metal-free solutions, porcelain-fused-to-metal restorations (PFM) are still widely used. Particularly for the latest ceramic systems, scarce information is present in the scientific literature about the ideal opaque layer thickness and the alloy color impact to achieve the desired final color of PFM restorations. This study aimed to evaluate the influence of opaque thickness variation layered on different metal alloys on the final color of PFM restorations. Opaque porcelain of one metal–ceramic system (VITA VM13) was layered in four different thicknesses (0.10 mm, 0.15 mm, 0.20 mm, and 0.25 mm) on three differently colored dental alloys: a gold–platinum alloy (yellowish), a gold–palladium alloy (light grayish), and a nickel–chromium alloy (dark grayish). The veneering porcelain layered over the opaque was kept constant (Base Dentine 0.45 mm, Transpa Dentine 0.50 mm, and Enamel 0.20 mm). Sixty specimens were fabricated, five samples for each combination of alloy/opaque thickness. The color difference (ΔE) between specimen and reference was measured using a clinical spectrophotometer. The two-way ANOVA revealed that the thickness of both the opaque (*p* < 0.001) and the metal alloy (*p* < 0.001) significantly influenced the ΔE values. Gray-colored alloys covered by a 0.10 mm thick opaque layer enabled the closest color match, whereas this occurred for yellow-color alloys covered by a 0.15 mm thick opaque layer. In contrast, the samples covered by a 0.25 mm thick opaque layer obtained the worst ΔE.

## 1. Introduction

One of the greatest challenges in restorative and prosthetic dentistry has always been imitating the appearance of natural teeth, in order to have a natural looking restoration that is functionally integrated in the mouth. In recent years, the interest in nonmetallic restorations increased, and new materials such as lithium disilicate, zirconia, and zirconia-reinforced lithium silicate were developed for this purpose. Notwithstanding this development, porcelain-fused-to-metal (PFM) restorations, combining wear resistance, strength, toughness, and reasonable aesthetics, are still considered a valid choice for dental prosthesis, especially for implant-supported restorations, particularly considering their high clinical success rate. Walton, in a survey, reported a 95.95% success rate at 14 years for implant-supported single crowns [1], a 92.75% success rate for tooth-supported crowns, and a 93.33% success for three-unit implant-supported bridges at 15 years [2].

Porcelain and ceramic materials have been used to produce aesthetic dental restorations since the early 1800s. Since that time, research and improvements in materials and techniques have dramatically increased the use of ceramic restorations [3]. However, the reproduction of a selected shade with dental porcelain is not an easy task [4]. Despite the availability of information concerning dental ceramic manufacturing, the importance of layering to achieve the best color match is often not addressed with a scientific rationale [5,6]. Douglas et al. [7] found that the ability to reproduce the color of a target shade tab differed among laboratories, and most of the crowns fabricated by these laboratories, when compared to the prescribed shade tab, were above the clinical threshold for an acceptable shade match. This was probably due to several factors influencing the final color of porcelain restorations. It is widely recognized that one of the most important factors is the thickness of different ceramic layers [8,9,10,11,12,13]. According to Barghi et al. [14], to produce an optimal color match, the thickness of opaque and body porcelain varies not only across shades but also between porcelain systems. Corciolani et al. [15] reported that, in the same ceramic system, the thickness of each ceramic layer and the ratio between the different layers significantly influence the final color of the PFM restorations.

Nevertheless, the problem is often managed subjectively, relying on the skill and experience of the individual ceramist involved.

In traditional PFM restorations, one of the reported manipulated variables dealing with aesthetics is the metal substrate. A wide number of alloys and metals are available for metal–ceramic use in dentistry. Each one has advantages and disadvantages, based primarily on its specific composition [16]. It has been widely reported that different alloys used in PFM restoration may influence the final color [17,18,19,20,21,22,23,24,25,26,27]. To minimize the effect of the metal substrate on the color of the restoration, an “opaque” porcelain layer is usually applied as a first ceramic layer. Some authors [19,24,28] found that variations of this opaque layer, particularly its thickness, influenced the final aesthetic results of the restorations. For this reason, the opaque plays an important role in the development of the shade and the aesthetic outcome of the PFM restorations [26,29,30]. Studies have been performed to identify the ideal thickness of the opaque layer [7,26,31] but nonrelevant information is available for the same assessment for last-generation ceramic systems. Considering these previous findings, the aim of this study was to test, by means of a spectrophotometric analysis, the influence of different opaque porcelain thicknesses layered on three metal alloys with different color on the final color of the PFM restorations.

Two different null hypotheses were tested:

(i) The thickness of the opaque layer applied on metal frameworks has no influence on the final color of PFM restorations.
(ii) The difference in color of metal frameworks has no influence on the final color of PFM restorations.

## 2. Materials and Methods

One metal ceramic system (Vita VM13, Vita Zahnfabrik, Germany) in 2M3 shade of the VITA Toothguide 3D-Master shade guide (Vita Zahnfabrik, Germany) was applied on three different dental alloys with different appearance in terms of color: a gold–platinum alloy (Esteticor Avenir-AL1, Cendres Métaux, Switzerland), yellowish shade; a gold–palladium alloy (Esteticor Plus-AL2, Cendres Métaux), light grayish shade; a nickel–chromium alloy (Biomate C-AL3, Silpo Srl, Italy), dark grayish shade (Table 1).

Since Barrett et al. [32] found no significant differences in shade matching accuracy between tooth-shaped tabs and discs, flat disc specimens were used in this study to facilitate the process of obtaining controlled thicknesses of the ceramic layers. Opaque porcelain was layered in four different thicknesses (0.10 mm—TH1, 0.15 mm—TH2, 0.20 mm—TH3, and 0.25 mm—TH4) in order to verify its masking ability. The thickness of the veneering porcelain was maintained constant (Base Dentine 0.45 mm, Transpa Dentine 0.50 mm, and Enamel 0.20 mm). Five samples were fabricated for each combination of alloy/opaque thickness, for a total of 60 specimens. Self-curing discs of acrylic resin (DuraLay; Reliance Dental Mfg Co, Worth, Ill), 0.3 mm in thickness and 15 mm in diameter, were prepared in a cylindrical, custom-made, stainless-steel mold. The mold allowed discs of fixed diameter (15 mm) and variable thickness to be fabricated. After placing the material into the mold, a glass plate was pressed onto the superficial layer to obtain a flat surface. Care was taken to avoid bubble formation within the resin. After polymerization, the resin specimen was extracted from the mold and placed in a refractory cast, which was then filled with investment (GC Stellavest; GC Europe NV, Leuven, Belgium) and placed in a burnout furnace (Ovomat 7; Manfredi Srl, Turin, Italy). At the end of the burnout cycle, the investment was moved to an induction-casting machine (Enterprise; Jelrus Intl, Melville, NY), and the metal alloys were cast. The resulting disc-shaped specimens were roughened with a sandblasting device (Skylab; Tecno-Gaz SpA, Parma, Italy) using 100 µm AlO_2_ particles (Ronvig Dental Mfg A/S, Daugaard, Denmark) at 4 bars of pressure. Following the manufacturer’s instructions [33], an “opaque paste” layer was applied and fired in a ceramic furnace (VITA Vacumat 4000 Premium T; VITA Zahnfabrik). By using the same mold as was used for fabricating the resin discs, it was possible to control the thickness of the ceramic applied before firing. After the application of each layer, the specimens were removed from the mold and fired following the manufacturer’s instructions (Table 2).

**Figure 1:**
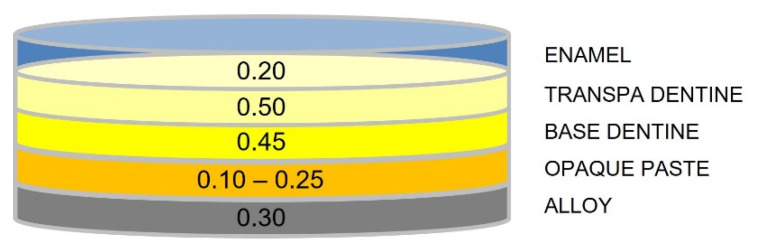
Specimen configuration (dimensions in mm).

Following application of the “opaque paste” layer, the “Base Dentin” layer, the “Traspa Dentin” layer, and the “Enamel” layer of the selected shade were independently added and fired. The tested combinations are reported in Table 3 and Figure 1.

**Figure 2:**
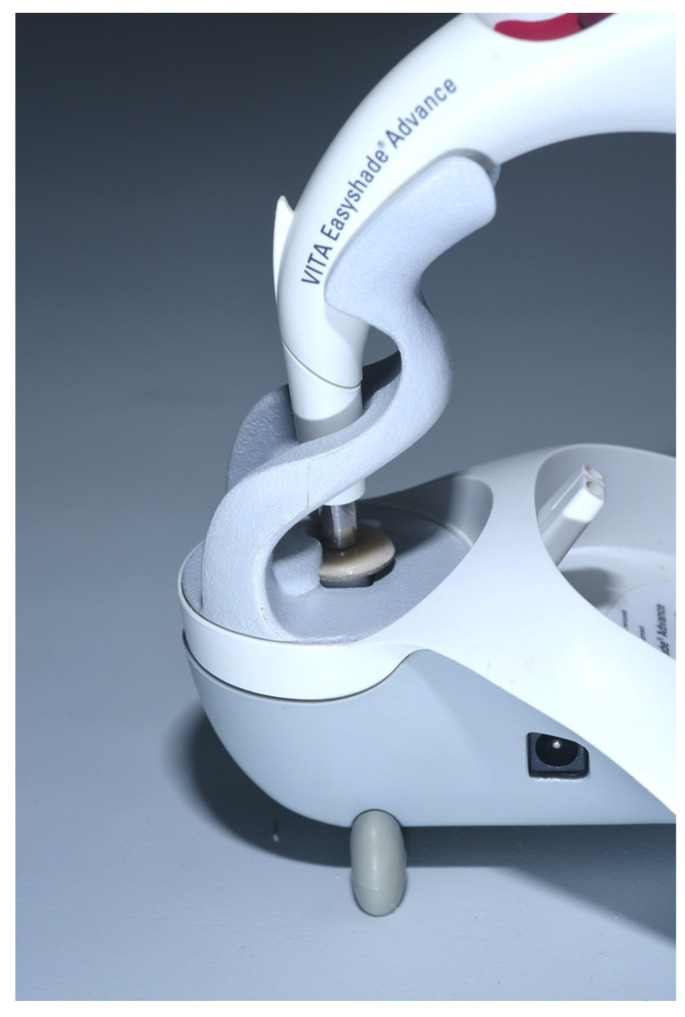
The clinical spectrophotometer VITA Easyshade fixed on a stand for color measurement.

Finally, each specimen was glaze-fired according to the manufacturer’s instructions. After each firing cycle, each layer was measured using an electronic digital caliper (1651 DGT; Beta Utensili Spa, Sovico, Italy) with an accuracy of 10 µm. The thickness of each layer was considered acceptable for the study only when the variation in thickness was ≤20 µm. For color assessment, spectrophotometric measurements were preferred to visual measurements, as they guarantee more reliable results when small color differences in dental ceramics are evaluated [34]. The color measurements were performed using the clinical spectrophotometer VITA Easyshade (VITA Zahnfabrik). The repeatability of color readings and the use of this clinical spectrophotometer were previously assessed [35,36]. The VITA Easyshade consists of a base unit and a handpiece. The color measurements were made by fixing the instrument on a stand (Figure 2), since it was shown that this arrangement resulted in a higher repeatability than freehand use [34]. All the measurements were performed while keeping the tip of the spectrophotometer perpendicular to the discs and in contact with the disc surface.

All measurements were consecutively performed after a single calibration process. The “restoration” mode on the spectrophotometer was selected and used throughout the study. The instrument, produced by the same manufacturer of the ceramic system and of the shade guide used in the present study, has stored in its memory the color coordinates of the VITA shades. In “restoration” mode, the instrument measures the color coordinates of the manufact (the specimen disc in this case), and then compares these color coordinates with the stored data. The VITA Easyshade calculates the differences in lightness (the perception by which white objects are distinguished from gray objects and light-colored objects are distinguished from dark-colored objects), chroma (the aspect of color in the Munsell color system according to which a specimen appears to differ from a gray of the same lightness or brightness, and which corresponds to saturation of the perceived color), hue (the attribute of color perception by means of which an object is judged to be red, yellow, green, etc.), and ΔE (the distance between points representing colors in the color space having rectangular coordinates). These differences are calculated by the spectrophotometer as a function of the E formula proposed by Clarke [37]. Although lightness, chroma, and hue are individually important to clarify the differences between the data stored in the device and the data measured from the specimens, ΔE is the most important parameter for calculating color differences [38]; for this reason, in the present study, ΔE values were statistically analyzed.

Having verified the normal distribution of the ΔE data according to the Kolmogorov–Smirnov test (*p* = 0.231) and the homogeneity of the group variances using the Levene test (*p* = 0.164), a two-way analysis of variance (ANOVA) was applied to determine the significance of the differences recorded for the groups. ΔE was considered to be the dependent variable, while alloys and opaque thicknesses were the factors. Statistical software (SPSS 16.0; SPSS, Inc, Chicago, Ill) was used for statistical analysis. The Tukey HSD test was used to perform multiple comparisons (α = 0.05).

## 3. Results

Table 2 reports the color differences between the intended shade and the specimen measured with the VITA Easyshade clinical spectrophotometer for VITA VM 13. Lower ΔE values mean a closer color match. The two-way ANOVA revealed that the metal alloy significantly influenced the ΔE values (*p* < 0.001). Specifically, regardless of the thickness of the opaque, the Tukey test revealed that the ΔE values for Esteticor Plus (mean of ΔE = 2.14) had significantly lower ΔE values than both Biomate C (mean of ΔE = 2.37) and Esteticor Avenir (mean of ΔE = 2.57).

The thickness of the opaque was a significant factor for ΔE values (*p* < 0.001). The Tukey HSD test for pairwise comparisons revealed that the overall ΔE values were lower for 0.10 mm (mean of ΔE = 2.24), 0.15 mm (mean of ΔE = 2.29), and 0.20 mm (mean of ΔE = 2.34) than for 0.25 mm (mean of ΔE = 2.57) (Table 4). The interaction between metal alloy and opaque thickness was found to be significant (*p* < 0.001).

## 4. Discussion

### 4.1. Opaque

The null hypothesis that no differences would be found among the different opaque thicknesses to mask the different metal frameworks was rejected, since final restorations with a 0.25 mm thick opaque layer performed significantly poorly in color matching the reference standard.

Although dental research has continuously improved the behavior and the performance of dental ceramics during the last 50 years, the main function that the opaque layer has to fulfill, that is masking the metal substrate, has never changed. In a previous study O’Connor et al. [31] investigated the castability, ceramic bond strength, and color stability after opaque application on different precious and nonprecious metals. No significant difference in color shades was reported when opaque layers of 0.2 mm thickness were achieved, even though no veneering porcelain was layered in that study. Terada et al. [19], with the aim of identifying the ideal opaque and body porcelain thickness for an acceptable appearance, reported that a minimally 0.3 mm thick opaque layer is required for gold-based alloys. Furthermore, they reported that the opaque layer did not have any effect on porcelain when the thickness was greater than 0.3 mm. Similarly to the previously cited paper by O’Connor et al. [31], in Terada et al. [19] veneering porcelain was not layered on top of the opaque porcelain.

On these bases, for the present study, five different opaque thicknesses were initially selected. They varied from 0.10 mm to 0.30 mm in 0.05 mm steps. After several attempts, the 0.30 mm groups were excluded due to the difficulty of firing a 0.30 mm thick opaque layer in a single firing process without creating cracks and bubbles in the opaque structure, thus introducing a bias for the study. Considering the elapsed time since the study of Terada et al. [19] was performed, the dental materials industry has significantly improved the quality of dental ceramics, and the manufacturer of the ceramic system tested in this study (VITA Zahnfabrik) currently recommends applying the opaque layer on the surface of the metal substructure “in a thin layer” [33]. Disregarding the alloy used, the results of the present study (Table 4) reaffirmed the manufacturer’s recommendation. Here, the closest color match was obtained with an opaque thickness of 0.10 mm (mean of ΔE = 2.24 ± 0.44), even though no statistically significant differences were found among TH1, TH2, and TH3 groups. Conversely, a statistically significant difference was found between these three groups and the TH4 group (0.25 mm), which performed slightly worse (mean of ΔE = 2.57 ± 0.21). These findings are not in agreement with those reported by Ozcelik et al. [24], where a 0.1 mm thick layer of opaque porcelain applied to various base metal alloys did allow not a reproducibility of the color of the opaque porcelain. In that study, the authors layered a 0.1 mm thick opaque layer on several base-metal alloys, and, by means of a colorimeter, they compared the color difference with a gold alloy chosen as standard. The differences between our findings and the data reported by O’ Connor et al. [31], Terada et al. [19], and Ozcelik et al. [24] can be ascribed, in addition to the different materials used, to the absence of the veneering porcelain on the opaque layer in those studies, whereas, in the present one, the veneering porcelain was added.

### 4.2. Alloy

The null hypothesis that different metal frameworks would have no influence on the final restoration color was rejected, since layering of porcelain on a light-gray alloy obtained the closest color match to reference standards.

Brewer et al. [39], by means of a spectrophotometric analysis, found that the resulting colorimetric values of porcelain fired on palladium–silver alloy differed significantly from those of porcelain fired on high-gold and nickel–chromium alloys, which were quite similar. Jacobs et al. [40] found that, for one porcelain shade, the color was shifted toward yellow/red for gold–palladium–platinum alloys in comparison to nickel–chromium and high-palladium alloys. Stevenson et al., in their review [41], reported that high-gold-backed samples were lighter (higher value) than samples backed by nickel chrome and silver–palladium alloys regardless of the tested shade. Gold-backed samples were found to be more yellow than base metals.

Although threshold ΔE values for perceptibility and acceptability cannot be taken as conclusive evidence, in general, the differences induced by the use of different alloys were perceptible, but at the limits for clinical acceptability, depending on the selected thresholds [42,43,44,45]. In the present study, two precious metal alloys (Au–Pt and Au–Pd) and one nonprecious metal alloy (Ni–Cr) were tested. Analyzing the data gathered from the spectrophotometric analysis, it can be stated that the Au–Pd alloy (Esteticor Plus-AL2) achieved the closest color match (mean ΔE = 2.14 ± 0.36) compared with AL3 (Biomate C-AL3) (mean ΔE = 2.37 ± 0.25) and Au–Pt alloy (Esteticor Avenir-AL1) (mean ΔE = 2.57 ± 0.22). To establish a general trend, for the present study, gray-colored alloys (AL2 and AL3) had a closer color match with standards when compared with a yellow-colored alloy (AL1).

Considering the interactions between alloys and opaque thicknesses, Esteticor Avenir (AL1) achieved the closest color match when a 0.15 mm thick opaque layer was tested (AL1-TH2; mean ΔE = 2.36 ± 0.15). Concerning Esteticor Plus, the best color match was obtained with a 0.10 mm thick opaque layer (AL2-TH1; mean ΔE = 1.84 ± 0.11). A similar trend was found with the use of Biomate C alloy (AL3-TH1; mean ΔE = 2.12 ± 0.27).

The authors are aware that testing a single shade from a single ceramic system, as well as choosing three representative colored alloy substructures from the wide variety available, cannot be regarded as conclusive evidence, which constitutes a limitation of the present study. Furthermore, the evaluation of the influence of the opaque layer was limited to color measurement, while adhesion and strength tests are advisable in future studies to improve the knowledge on the behavior of the opaque layer in relation to thickness. It should also be noted that several other factors might affect the final shade, such as the firing process, which might induce changes depending on the different alloys used. Moreover, this study had the limitation common to all in vitro studies that the actual clinical conditions could not be replicated, such as the temperature fluctuations in the oral cavity that might affect the color stability of the ceramic, although no evidence has been identified at present in this regard [46,47].

Notwithstanding these limitations, the outcomes of this research provide some evidences and highlight the importance of understanding how each layer of ceramic systems behaves and influences achieving the closest color match.

## 5. Conclusions

Within the limitations of this study, for the ceramic system and the casting alloys tested, the following conclusions can be drawn:

(a) The Vita VM13 veneering ceramic in 2M3 shade with a thin opaque layer (0.10–0.15 mm) achieved the closest color match with the standards.

(b) Gray-colored alloys required an opaque layer of 0.10 mm thickness to grant the closest color match, while the yellow-colored alloy achieved the closest color match with a 0.15 mm thick opaque layer.

(c) Layering still plays a determinant role in achieving the color match with the intended shade.

## Figures and Tables

**Table 1:** Composition and characteristic of the alloys used as framework.

Alloy	Chemical Composition	Manufacturer	Color
Esteticor Avenir (AL1)	Au 84.0%, Pt 10.9%, Pd 2.4%, Ag 0.2%, Zn 2.2%, Ir 0.1%, Fe 0.2%	Cendres Métaux	Yellow
Esteticor Plus (AL2)	Au 45.0%, Pd 38.9%, Ag 5.0%, In 8.6%, Ga 1.4%, Sn 0.5%, Cu 0.4%, Ru 0.2%	Cendres Métaux	Light gray
Biomate C (AL3)	Ni 62.0%, Cr 25.0%, Mo 9.5%, Si 3.3% Others (P, S, Cu, Al, V, Nb)	Silpo	Dark gray

**Table 2:** Firing chart.

	Pre-Drying Temperature (°C)	Pre-Drying Time (min)	Heating Time (min)	Temperature Raise Time (°C/min)	End Temperature (°C)	Hold Time End Temperature (min)	Vacuum Holding Time (min)
Oxidation	According to Manufacturer instructions						
Opaque Paste P *	500	4.00	5.12	75	890	1.00	5.12
Opaque Paste NP *	500	4.00	5.36	75	920	1.00	5.36
Base Dentine	500	6.00	6.55	55	880	1.00	6.55
Traspa Dentine	500	6.00	6.44	55	870	1.00	6.44
Enamel	500	4.00	4.45	80	880	1.00	-

P = precious alloy; NP = non-precious alloy; * depending on the alloy

**Table 3:** Thicknesses of the combination tested (mm).

Opaque Paste	Base Dentine	Transpa Dentine	Enamel	Total Ceramic	Total Specimen
0.10	0.45	0.50	0.20	1.15	1.45
0.15				1.20	1.50
0.20				1.25	1.55
0.25				1.30	1.60

**Table 4:** Vita Easyshade color evaluation of the VM13 ceramic in 2M3 shade specimens. Measurements were performed in “restoration” mode. ΔE values represent the differences with standard color parameters for the selected shade set in the instrument. In the interaction column and row, different letters significant differences when interactions were assessed at the univariate level.

	0.10 mm (TH1)	0.15 mm (TH2)	0.20 mm (TH3)	0.25 mm (TH4)	Total for Alloy	Alloys Interactions
**Avenir (AL1)**	2.76 (0.15)	2.36 (0.15)	2.58 (0.26)	2.56 (0.15)	2.57 (0.22)	C
**Plus (AL2)**	1.84 (0.11)	2.04 (0.23)	2.08 (0.25)	2.60 (0.28)	2.14 (0.36)	A
**Biomate (AL3)**	2.12 (0.27)	2.46 (0.11)	2.36 (0.18)	2.54 (0.21)	2.37 (0.25)	B
Total for opaque thickness	2.24 (0.44)	2.29 (0.24)	2.34 (0.30)	2.57 (0.21)		
Thicknesses Interactions	a	a	a	B		

## Data Availability

The data presented in this study are available on request from the corresponding author. The data are not publicly available due to the university’s policy on access.

## References

[1] Walton T.R. (2016). The Up-to-14-Year Survival and Complication Burden of 256 TiUnite Implants Supporting One-Piece Cast Abutment/Metal-Ceramic Implant-Supported Single Crowns. J. Oral. Maxillofac. Implant..

[2] Walton T.R. (2015). An Up-to-15-Year Comparison of the Survival and Complication Burden of Three-Unit Tooth-Supported Fixed Dental Prostheses and Implant-Supported Single Crowns. J. Oral. Maxillofac. Implant..

[3] Bayne S.C., Ferracane J.L., Marshall G.W., Marshall S.J., van Noort R. (2019). The Evolution of Dental Materials over the Past Century: Silver and Gold to Tooth Color and Beyond. J. Dent. Res..

[4] Fani G., Vichi A., Davidson C.L. (2007). Spectrophotometric and visual shade measurements of human teeth using three shade guides. Am. J. Dent..

[5] Kelly J.R., Nishimura I., Campbell S.D. (1996). Ceramics in dentistry: Historical roots and current perspectives. J. Prosthet. Dent..

[6] Vichi A., Fraioli A., Davidson C.L., Ferrari M. (2007). Influence of thickness on color in multi-layering technique. Dent. Mater..

[7] Douglas R.D., Brewer J.D. (2003). Variability of porcelain color reproduction by commercial laboratories. J. Prosthet. Dent..

[8] Ozturk O., Uludag B., Usumez A., Sahin V., Celik G. (2008). The effect of ceramic thickness and number of firings on the color of two all-ceramic systems. J. Prosthet. Dent..

[9] Corciolani G., Vichi A., Goracci C., Ferrari M. (2009). Colour correspondence of a single ceramic system in two different shade guides. J. Dent..

[10] Fazi G., Vichi A., Corciolani G., Ferrari M. (2009). Spectrophotometric evaluation of color match to VITA Classical shade guide of four different porcelain systems for metal-ceramic restorations. Am. J. Dent..

[11] Corciolani G., Vichi A., Louca C., Ferrari M. (2011). Color match of two different ceramic systems to selected shades of one shade guide. J. Prosthet. Dent..

[12] Kürklü D., Azer S.S., Yilmaz B., Johnston W.M. (2013). Porcelain thickness and cement shade effects on the colour and translucency of porcelain veneering materials. J. Dent..

[13] Xu B. (2021). Effects of dentin and enamel porcelain layer thickness on the color of various ceramic restorations. J. Esthet. Restor. Dent..

[14] Barghi N., Lorenzana R.E. (1982). Optimum thickness of opaque and body porcelain. J. Prosthet. Dent..

[15] Corciolani G., Vichi A., Louca C., Ferrari M. (2010). Influence of layering thickness on the color parameters of a ceramic system. Dent. Mater..

[16] Roberts H.W., Berzins D.W., Moore K., Charlton D.G. (2009). Metal–ceramic alloys in dentistry: A review. J. Prosthodont..

[17] O’Neal S.J., Leinfelder K.F., Lemons J.E., Jamison H.C. (1987). Effect of metal surfacing on the color characteristics of porcelain veneer. Dent. Mater..

[18] Seghi R.R., Johnston W.M., O’Brien W.J. (1986). Spectrophotometric analysis of color differences between porcelain systems. J. Prosthet. Dent..

[19] Terada Y., Sakai T., Hirayasu R. (1989). The masking ability of an opaque porcelain: A spectrophotometric study. Int. J. Prosthodont..

[20] Woolsey G.D., Johnson W.M., O’Brien W.J. (1984). Masking power of dental opaque porcelains. J. Dent. Res..

[21] Crispin B.J., Seghi R.R., Globe H. (1991). Effect of different metal ceramic alloys on the color of opaque and dentin porcelain. J. Prosthet. Dent..

[22] Stavridakis M.M., Papazoglou E., Seghi R.R., Johnston W.M., Brantley W.A. (2000). Effect of different high-palladium metal-ceramic alloys on the color of opaque porcelain. J. Prosthodont..

[23] Stavridakis M.M., Papazoglou E., Seghi R.R., Johnston W.M., Brantley W.A. (2004). Effect of different high-palladium metal–ceramic alloys on the color of opaque and dentin porcelain. J. Prosthet. Dent..

[24] Ozcelik T.B., Yilmaz B., Ozcan I., Kircelli C. (2008). Colorimetric analysis of opaque porcelain fired to different base metal alloys used in metal ceramic restorations. J. Prosthet. Dent..

[25] Vichi A., Louca C., Corciolani G., Ferrari M. (2011). Color related to ceramic and zirconia restorations: A review. Dent. Mater..

[26] Arif R., Yilmaz B., Mortazavi A., Ozcelik T.B., Johnston W.M. (2018). Effect of metal opaquer on the final color of 3 ceramic crown types on 3 abutment configurations. J. Prosthet. Dent..

[27] Jörn D., Waddell N., Swain M.W. (2010). The influence of opaque application methods on the bond strength and final shade of PFM restorations. J. Dent..

[28] Terada Y., Maeyama S., Hirasu R. (1989). The influence of different thicknesses of dentin porcelain on the color reflected from thin opaque porcelain fused to metal. Int. J. Prosthodont..

[29] Shillingburg H.T., Hobo S., Whitsett L.D., Jacobi R., Brackett S.E. (1997). Fundamentals of Fixed Prosthodontics.

[30] Wataha J.C. (2002). Alloys for prosthodontic restorations. J. Prosthet. Dent..

[31] O’Connor R.P., Mackert J.R., Myers M.L., Parry E.E. (1996). Castability, opaque masking, and porcelain bonding of 17 porcelain-fused-to-metal alloys. J. Prosthet. Dent..

[32] Barrett A.A., Grimaudo N.J., Anusavice K.J., Yang M.C.K. (2002). Influence of tab and disk design on shade matching of dental porcelain. J. Prosthet. Dent..

[33] Vita (2007). VM13 Working Instruction, revised ed..

[34] Seghi R.R., Hewlett E.R., Kim J. (1989). Visual and instrumental colorimetric assessment of small color differences on translucent dental porcelain. J. Dent. Res..

[35] Corciolani G., Vichi A. (2006). Repeatability of color reading with a clinical and a laboratory spectrophotometer. Int. Dent. S. Afr..

[36] Lehmann K.M., Igiel C., Schmidtmann I., Scheller H. (2010). Four color-measuring devices compared with a spectrophotometric reference system. J. Dent..

[37] Clarke F.J.J. (1983). Measurement of the Color of Human Teeth. Dental Ceramics: Proceedings of the First International Symposium on Ceramics.

[38] CIE (Commission Internationale de l’Eclairage) (1986). Colorimetry—Technical Report.

[39] Brewer J.D., Akers C.K., Garlapo D.A., Sorensen S.E. (1985). Spectrometric analysis of the influence of metal substrates on the color of metal–ceramic restorations. J. Dent. Res..

[40] Jacobs S.H., Goodacre C.J., Moore B.K., Dykema R.W. (1987). Effect of porcelain thickness and type of metal–ceramic alloy on color. J. Prosthet. Dent..

[41] Stevenson B., Ibbetson R. (2010). The effect of the substructure on the colour of samples/restorations veneered with ceramic: A literature review. J. Dent..

[42] Vichi A., Ferrari M., Davidson C.L. (2004). Color and opacity variations in three different resin-based composite products after water aging. Dent. Mater..

[43] Ghinea R., Pérez M.M., Herrera L.J., Rivas M.J., Yebra A., Paravina R.D. (2010). Color difference thresholds in dental ceramics. J. Dent..

[44] Paravina R.D., Ghinea R., Herrera L.J., Bona A.D., Igiel C., Linninger M., Sakai M., Takahashi H., Tashkandi E., Perez Mdel M. (2015). Color difference thresholds in dentistry. J. Esthet. Restor. Dent..

[45] Paravina R.D., Pérez M.M., Ghinea R. (2019). Acceptability and perceptibility thresholds in dentistry: A comprehensive review of clinical and research applications. J. Esthet. Restor. Dent..

[46] Heydecke G., Zhang F., Razzoog M.E. (2001). In vitro color stability of double-layers veneers after accelerated aging. J. Prosthet. Dent..

[47] De Carvalho Panzeri Pires-de-Souza F., Assirati Casimiro L., da Fonseca Roberti Garcia L., Rodrigues Cruvinel D. (2009). Color stability of dental ceramics submitted to artificial accelerated aging after repeated firings. J. Prosthet. Dent..

